# Supplementary material for: Glucocorticoid Nanoparticles Show Full Therapeutic Efficacy in a Mouse Model of Acute Lung Injury and Concomitantly Reduce Adverse Effects
Source: Int J Mol Sci. 2023 Nov 28;24(23):16843. doi: 10.3390/ijms242316843 (PMC10705980; doi:10.3390/ijms242316843)
Supplement: Supplementary file 1 [file ijms-24-16843-s001.zip › ijms-2675689-supplementary.pdf]

Supplementary Information (SI)

1. Synthesis and characterization of BNP

$[ZrO]^{2+}[(BMP)_{0.9}(FMN)_{0.1}]^{2-}$ IOH-NP (designated BNP) were developed as part of a new platform of nanomaterials for drug delivery (BMP: betamethasone phosphate; FMN: flavine mononucleotide) [S1]. Generally, IOH-NP are characterized by a saline composition with an inorganic cation and a drug anion, which is functionalized by phosphate, sulfonate, or carboxylate groups [S2].

BNP synthesis and characterization have already been published [S3]. In brief, they were prepared by injection of aqueous $ZrOCl_2 \times 8H_2O$ into an aqueous solution of $Na_2(BMP)$ and $Na(HFMN) \times 2H_2O$ (Supplementary Figure S1A). After purification, colloidally highly stable suspensions (1-5 mg/ml) were obtained that do not show any sedimentation over several weeks. Particle size, size distribution, and zeta-potential were examined by dynamic light scattering (DLS) and scanning electron microscopy (SEM). Accordingly, a hydrodynamic diameter of 40-90 nm (DLS) and a diameter of 31±9 nm (SEM, calculated by statistical evaluation of >100 particles on SEM images) were obtained with a negative surface charging (–40 to –30 mV at pH 5 to 9) (Supplementary Figure S1B-D) [S3]. The larger particle diameter from DLS reflects the hydrodynamic diameter and the presence of a rigid layer of water molecules adsorbed on the particle surface [S2,S3]. The chemical composition of BNP was validated by different methods, including X-ray diffraction (XRD), energy-dispersive electron spectroscopy (EDXS), Fourier-transform infrared (FT-IR) spectroscopy, elemental analysis (EA, C/H/N/S), and total organics combustion/thermogravimetry (TG) [S3]. Whereas the presence of Zr and BMP/FMN were qualitatively confirmed by EDXS (Zr, P) and FT-IR (BMP, FMN), the cation-to-anion ratio and the overall composition were quantified by EA and TG. In sum, the composition of BNP carrying only the drug was confirmed with a BMP-load of 81.5 % of the total IOH-NP mass (18.5 % due to $[ZrO]^{2+}$ as inorganic cation) [S3]. After the addition of 10 mol-% of FMN, BNP show intense green emission with a maximum at 530 nm (Supplementary Figure S1E). They can be excited in a range of 250-510 nm [S3].

2. Inductive coupled plasma mass spectrometry (ICP-MS)

BNP containing 0.2 mg of Zr were administered to wildtype mice by i.p. injection (total volume: 500 µl), and 5 hrs later the mice were sacrificed [S4]. The left and caudate liver lobes, a central 20 cm piece of the small intestine, and the entire lung were collected, washed twice with PBS, weighted and stored at −80°C. The tissue samples were analyzed by a commercial microanalytical laboratory (Labor Pascher, Remagen, Germany) using ICP-MS. To this end, approx. 100 mg of the provided material were dissolved overnight in a PFA-pressure system with HNO_3/HF at 180°C [S4]. Following the addition of Rh and Ir as internal standards, Zr was detected using an iCAPTM Q ICP-MS instrument (Thermo Fisher Scientific, Waltham, MA, USA).

[S1] M. Ischyropoulou, K. Sabljo, L Schneider, C. M. Niemeyer, J. Napp, C. Feldmann, F. Alves, *Adv. Mater.* **2023**, 2305151(1-15); M. Khorenko, A. Meschkov, J. Napp, J. Pfeifer, J. Stier, F. Alves, U. Schepers, C. Feldmann, *J. Mater. Chem. B* **2023**, *11*, 3635-3649; V. Rein, E. Zittel, K. Hagens, N. Redinger, U. Schepers, H. Mehlhorn, U. Schaible, C. Feldmann, *Adv. Funct. Mater.* **2019**, *29*, 1900543(1-11); M. Poß, E. Zittel, C. Seidl, A. Meschkov, L. Muñoz, U. Schepers, C. Feldmann, *Adv. Funct. Mater.* **2018**, *28*, 1801074(1-8).

[S2] B. L. Neumeier, M. Khorenko, F. Alves, O. Goldmann, J. Napp, U. Schepers, H. M. Reichardt, C. Feldmann, *ChemNanoMat* **2019**, *5*, 24-45.

[S3] J. Napp, M. A. Markus, J. G. Heck, C. Dullin, W. Möbius, D. Gorpas, C. Feldmann, F. Alves, *Theranost.* **2018**, *8*, 6367-6368; J. G. Heck, J. Napp, S. Simonato, J. Möllmer, M. Lange, H. R. Reichardt, R. Staudt, F. Alves, C. Feldmann, *J. Am. Chem. Soc.* **2015**, *137*, 7329-7336.

[S4] J. Kunze, S. Koelling, M. Reich, M. A. Wimmer, *Fresenius J. Anal. Chem.* **2000**, *366*, 165-166; T. K. Kaiser, M. Khorenko, A. Moussavi, M. Engelke, S. Boretius, C. Feldmann, H. M. Reichardt, *J. Control Release* **2020**, *319*, 360-370.

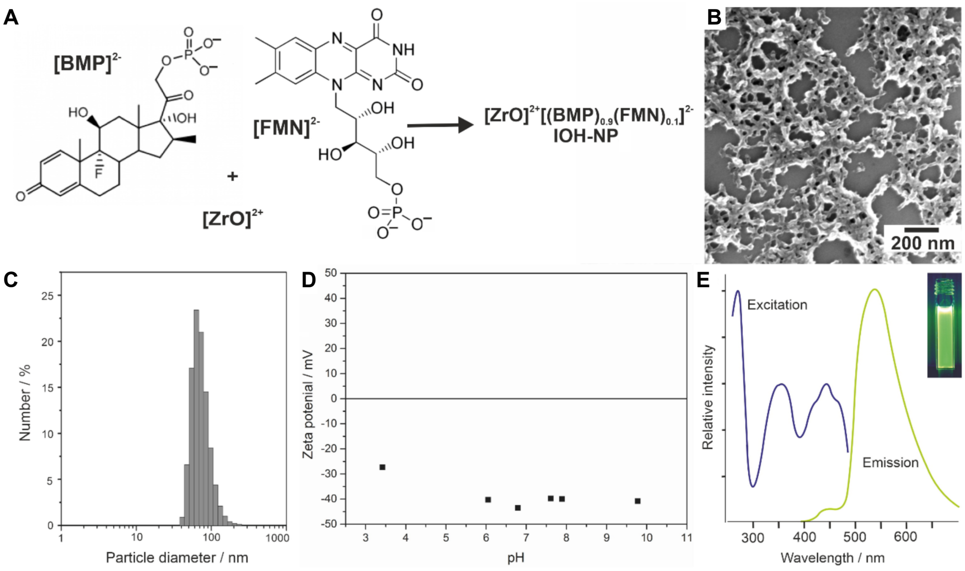

Supplementary Figure S1. Synthesis and characterization of $[ZrO]^{2+}[(BMP)_{0.9}(FMN)_{0.1}]^{2-}$ IOH-NP (designated BNP). **(A)** Scheme illustrating the aqueous synthesis, **(B)** SEM image, **(C)** particle size distribution according to DLS, **(D)** zeta-potential analysis (in water), **(E)** excitation and emission spectra with photo of aqueous suspension with green emission (upon excitation at 366 nm). [S3]

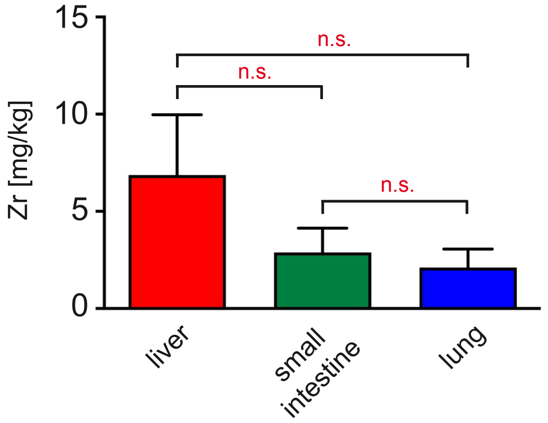

Supplementary Figure S2. Concentration of BNP in different organs of mice. Wildtype mice were i.p. injected with 500 µl of BNP containing 0.2 mg of Zr and 5 hrs later liver, small intestine and lung were dissected. The relative Zr concentration was analyzed by inductive coupled plasma mass spectrometry (ICP-MS) and is depicted as the mean amount of Zr per weight of wet tissue ± SEM (N = 4). Statistical analysis was achieved by One-way ANOVA followed by Newman-Keuls multiple comparison test.

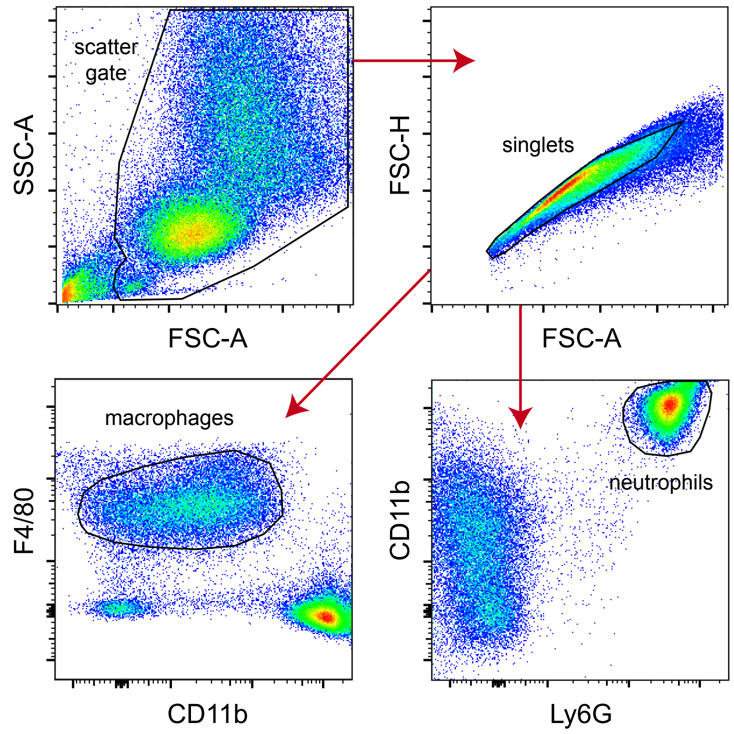

Supplementary Figure S3. Exemplary flow cytometric analysis of BALF cells collected from a mouse 15 hours after injection with LPS and OA. Relevant cells were first defined on the basis of their forward and sideward scatter, followed by gating on singlets. Macrophages were then identified using antibodies recognizing CD11b and F4/80, and neutrophils using antibodies recognizing CD11b and Ly6G.

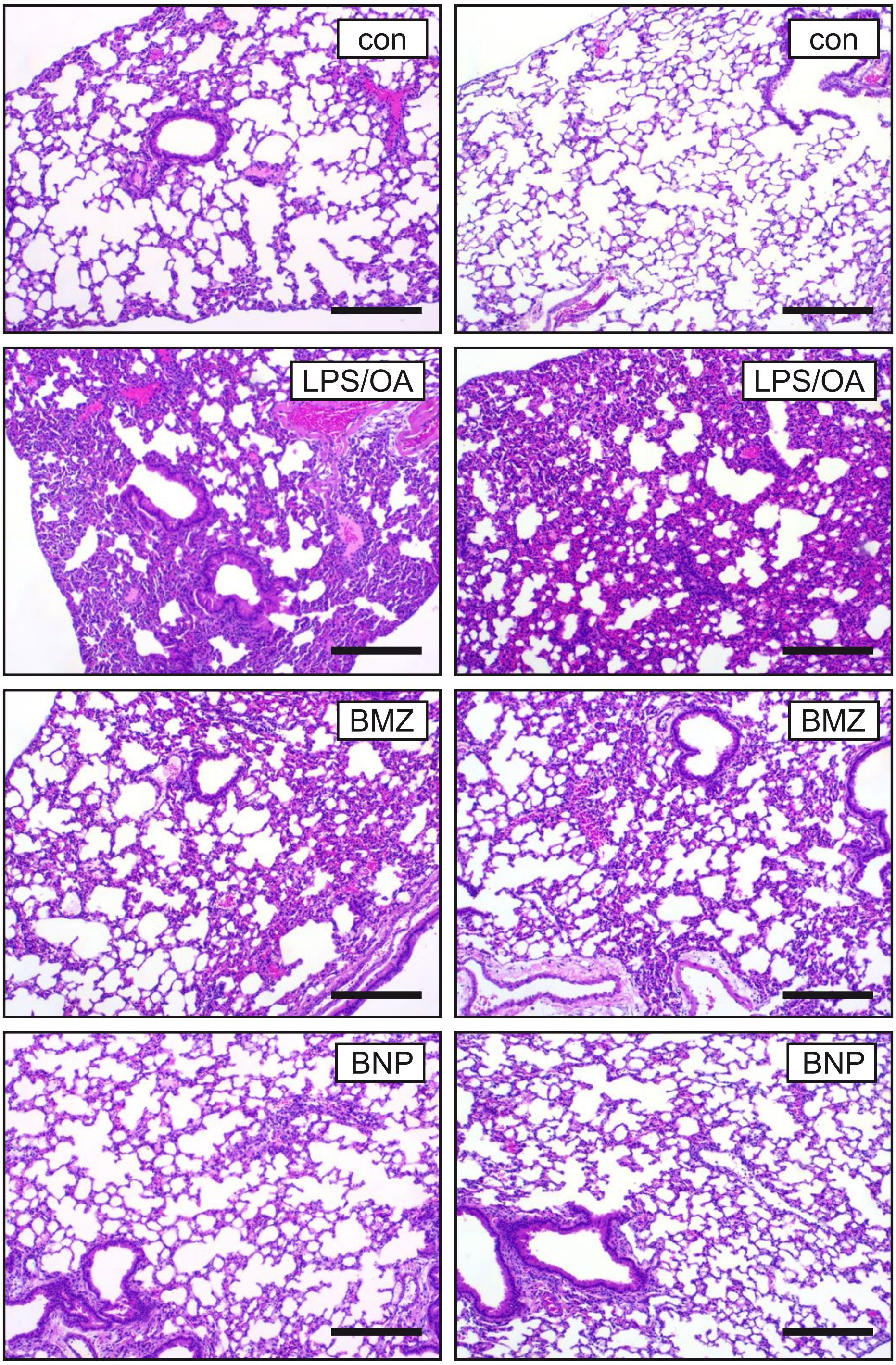

Supplementary Figure S4. Histological analysis of the lung in a mouse model of ALI. C57BL/6 mice were injected with LPS and OA followed by treatment with BMZ or BNP. The lung was fixed in PFA followed by H&E staining. Two additional sections from the experiment shown in Figure 3 are depicted for each condition. The size bar corresponds to 500 μm.
